# *Staphylococcus haemolyticus* is a reservoir of antibiotic resistance genes in the preterm infant gut

**DOI:** 10.1080/19490976.2025.2519700

**Published:** 2025-06-22

**Authors:** Lisa E. Lamberte, Elizabeth M. Darby, Raymond Kiu, Robert A. Moran, Antia Acuna-Gonzalez, Kathleen Sim, Alexander G. Shaw, J. Simon Kroll, Gusztav Belteki, Paul Clarke, Heather Felgate, Mark A. Webber, William Rowe, Lindsay J. Hall, Willem Van Schaik

**Affiliations:** a Institute of Microbiology and Infection, University of Birmingham, Birmingham, UK; b Department of Microbes, Infection and Microbiomes, School of Infection, Inflammation and Immunology, College of Medicine and Health, University of Birmingham, Birmingham, UK; c Quadram Institute Bioscience, Norwich Research Park, Norwich, UK; d Faculty of Medicine, Imperial College London, London, UK; e The Rosie Hospital, Cambridge University Hospitals NHS Foundation Trust, Cambridge, UK; f Neonatal Intensive Care Unit, Norfolk and Norwich University Hospitals NHS Foundation Trust, Norwich, UK; g Norwich Medical School, University of East Anglia, Norwich, UK

**Keywords:** *Staphylococcus*, neonates, antibiotic resistance, microbial genomics, microbial evolution, gut microbiota

## Abstract

*Staphylococcus haemolyticus* is an important cause of sepsis in preterm infants, with gut colonization being recognized as a risk factor for infection. To better understand the diversity of *S. haemolyticus* among preterm infants, we generated genome sequences of *S. haemolyticus* strains (*n* = 140) from 44 stool samples of 22 preterm infants from four hospitals in England. Core genome phylogenetic analyses, incorporating 126 publicly available *S. haemolyticus* genome sequences, showed that 85/140 (60.1%) of the isolates, from three different hospitals, formed a clonal group with 78/85 (91.7%) strains having Multi-Locus Sequence Type (ST) 49. Antibiotic resistance genes were prevalent in the genomes. There was a strong association between the presence of *mecA* and phenotypic resistance to oxacillin, and the *aacA-aphD* gene and phenotypic resistance to gentamicin. While *mecA* was near-ubiquitous, none of the strains from the preterm infant cohort had a complete Staphylococcal Cassette Chromosome *mec* (SCC*mec*) element. The *aacA-aphD* gene was associated with the transposon Tn*4001* in multiple chromosomal and plasmid contexts. Our data suggest the existence of a distinct sub-population of *S. haemolyticus* that has adapted to colonize the gut of preterm infants, and widespread horizontal gene transfer and recombination among this frequent colonizer of the preterm infant gut.

## Introduction

Preterm infants, defined as neonates born before 37 weeks of gestation, are a patient population that is particularly vulnerable to life-threatening complications. There are approximately 15 million preterm births globally, with an estimated 0.66 million deaths associated with preterm birth.^1,2^ The heightened vulnerability of preterm infants in neonatal intensive care units (NICUs) to nosocomial infections arises from their compromised immune defenses, prolonged use of invasive medical tools, extended hospital stays, and concurrent medical complexities.^3^ Late-onset sepsis (LOS) is a significant cause of morbidity and mortality among preterm infants, with coagulase-negative staphylococci (CoNS) as one of the leading causative agents of these infections.^4–6^ Among CoNS, *Staphylococcus haemolyticus*, a skin commensal that commonly colonizes the neonatal gut, is a prominent cause of LOS in preterm infants.^7–9^ To treat neonatal sepsis, current UK guidelines recommend the use of a combination of antibiotics of multiple classes, primarily a β-lactam antibiotic (benzyl-penicillin or flucloxacillin) and the aminoglycoside gentamicin.^10^ Among preterm infants hospitalized in UK NICUs between 2010 and 2017, 77% receive antibiotics at least once, with benzyl-penicillin and gentamicin being the most used antibiotics in these infants.^11^

The emergence of multidrug-resistant strains among hospital-acquired pathogens in NICU settings poses a substantial challenge to treatment strategies and infant care.^12,13^ High-resolution genomic studies can provide a deep understanding of the intricate dynamics of these pathogens, shedding light on colonization patterns and the evolution of resistance mechanisms over time.^14–18^ While *S. haemolyticus* is one of the most common CoNS causing human infections, it remains understudied and we thus lack an understanding of its diversity and potential for transmission in healthcare systems.

In this study, we employed high-throughput whole-genome sequencing to investigate the genomic diversity of *S. haemolyticus* that colonized the gut of preterm infants that were hospitalized in UK NICUs. We specifically focussed on its antibiotic resistance genes and the mobile genetic elements these genes are carried on, to provide insights into the threat posed by *S. haemolyticus* to critically ill preterm infants. Our findings can inform targeted strategies for better patient care and the management of neonatal infections, thus contributing to efforts that enhance clinical outcomes for vulnerable neonates.

## Materials and methods

### *Abundance and prevalence of* S. haemolyticus *in the preterm infant gut*

We used the results of an earlier study in which 16S rRNA gene profiling was performed to characterize the gut microbiome of 497 preterm infants as part of the Baby-Associated Microbiota of the Intestine (BAMBI) study (European Nucleotide Archive Accession Number: PRJEB31653)^19^ to estimate *S. haemolyticus* prevalence and abundance in the preterm infant gut. Sample details, including the proportion of reads assigned to Operational Taxonomic Units (OTUs) putatively equivalent to *S. haemolyticus*, as defined in,^19^ were extracted for analysis. Data visualization and figure generation were performed using RStudio (version 4.4.1), with the ggplot2 package (version 3.5.1).

### *Isolation of* S. haemolyticus *from preterm infant stool samples*

*S. haemolyticus* strains were isolated from preterm infant stool samples that were part of the Baby-Associated Microbiota of Intestine (BAMBI) study^19^ and the NeoM study.^20^ A total of 140 isolates were collected from 44 stool samples of 22 infants. These stool samples originated from four hospitals in the East of England, specifically in Norwich; *n* = 28), two hospitals in London (London 1; *n* = 12; London 2; *n* = 3), and Cambridge (*n* = 1). Metadata of the samples from which *S. haemolyticus* was isolated are provided in Table S1. Isolates were selectively cultured from stool samples using mannitol salt agar (Oxoid), on which *S. haemolyticus* forms pink colonies, and subsequently, on Tryptic Soy Agar (Thermo Scientific) +5% sheep blood (Blood Agar), on which *S. haemolyticus* can be presumptively identified by β-hemolysis. A total of 5 colonies per stool sample were collected and prepared for whole genome sequencing.

### Genomic DNA extraction and whole genome sequencing

Isolates that were sent for whole genome sequencing were grown from a single colony in Tryptic Soy Broth (Oxoid) for 18 h at 37°C. Genomic DNA for short-read (Illumina) sequencing was extracted using the Wizard Genomic DNA Purification Kit (Promega) according to the manufacturer’s instructions with a pretreatment of pelleted cells in 600 µl 50 mm EDTA with 1 mg/ml lysozyme and 1 mg/ml lysostaphin for 30 min at 37°C. Genomic DNA for long-read (Oxford Nanopore Technologies) sequencing was extracted using the Monarch HMW DNA Extraction Kit for Tissue (New England Biolabs) according to the manufacturer’s instructions with the addition of lysozyme and lysostaphin as above, prior to cell lysis. For short-read sequencing, DNA libraries were run at a final concentration of 1.5 pM, which included a 1% PhiX spike-in (PhiX Control v3 Illumina Catalogue FC-110–3001) on an Illumina Nextseq500 system using Mid Output Flowcells (NSQ® 500 Mid Output KT v2 (300 CYS) Illumina Catalogue FC-404–2003). For long-read sequencing, DNA libraries were prepared using the ligation sequencing kit SQK-LSK109 (Oxford Nanopore Technologies) and sequenced on the MinION using R9.4.1 flow cells, according to the manufacturer’s instructions.

### Genome assembly

For short-read data, adapters were removed and trimmed using fastp (v.0.23.2).^21^ The short-read sequences were then assembled using SPAdes (v.3.14.1) with default parameters applied.^22^ DNA assemblies were then annotated using Prokka (v.1.14.6)^23^ and the sequence type (ST) was assigned using mlst (v.2.16.1),^24^ using the PubMLST database.^25^ *Staphylococcus haemolyticus* type strain NCTC 11,042 was used as a control strain and was processed alongside the stool sample isolates to benchmark the sequencing and assembly workflow. Scaffold assemblies were evaluated using QUAST (v5.0.2),^26^ and isolates with genome coverage below 10x were excluded from downstream analyses. To further assess genome quality and confirm species identity, assemblies were analyzed using FastANI (v1.1).^27^ Isolates with an average nucleotide identity (ANI) below 95% were removed from the final dataset.

The long-read data was basecalled using Guppy (v.0.1.0).^28^ The quality of the data was examined using NanoStat (v.1.6.0).^29^ Filtlong (v.0.2.1)^30^ was used to filter out any reads shorter than 1 kbp and exclude the worst 5% of the reads, using the option – keep_percent 95. Hybrid assemblies using the combination of long- and short-read data were generated using Unicycler (v.0.4.7).^31^ When Unicycler was unable to generate a complete assembly, a long-read-first assembly strategy was adopted, using Flye (v.2.9.1-b1780)^32,33^ to assemble the long-read dataset, which was then integrated into the Unicycler pipeline to enhance the assembly process. SnapGene (www.snapgene.com) (v5.2.4) was used to visualize plasmids.

### Genome sequence analyses

*S. haemolyticus* genomes from Cavanagh et al.^14^ and the genome sequence of the *S. haemolyticus* type strain NCTC11042 (Genbank: GCF_900458595.1) were added to our dataset. Roary (v.3.12.0)^34^ was used to generate a core gene alignment, which was then used to infer a maximum likelihood (ML) phylogeny, using RAxML (v.1.1.0) with the GTR+G model with 100 bootstrap replicates.^35^ Recombination events were removed using ClonalFrameML (v.1.12).^36^ snp-dists (v0.8.2) (https://github.com/tseemann/snp-dists) was used to quantify the number of SNPs in core genome alignments. *fastbaps*^37^ was used to identify clusters within the *S. haemolyticus* population. Assemblies were searched for the presence of antibiotic resistance genes using ABRicate (v.1.0.1)^38^ with the CARD database (v3.1.1)^39^ and plasmid replicon sequences with the PlasmidFinder database (v2.0.1)^40^ using cutoffs of >80% coverage and >80% identity. MOB-suite (v3.0.3)^41^ was used to predict transferability of plasmids. Phylogenetic trees were visualized and annotated using iTOL.^42^ SCC*mec* typing was performed using SCC*mec*Finder.^43^ To further assess the presence of SCC*mec*-encoded elements, a reference dataset comprising 112 SCC*mec* elements, representing SCC*mec* types I to XIV, was compiled (Table S2). Each *S. haemolyticus* assembly from our study collection was compared against this reference database using BLASTn.^44^ SCC*mec* elements were identified based on >80% sequence coverage and >80% nucleotide identity.

### Antibiotic susceptibility testing

Antibiotic susceptibility testing was performed on 58 *S. haemolyticus* isolates from the BAMBI collection, using the broth microdilution method^45^ and the antibiotics oxacillin and gentamicin (both obtained from Merck Life Science UK Ltd), and the results were interpreted according to EUCAST breakpoints.^46^ *S. haemolyticus* NCTC11042 was used as a control. The minimum inhibitory concentration (MIC) was determined as the mode of three biological replicates. We also incorporated antibiotic susceptibility data from 123 *S. haemolyticus* strains from a study by Cavanagh and colleagues.^14^

### Statistical analyses

All statistical analyses were performed using R version 4.2.2. To investigate the association between the presence of genes and phenotypic resistance, a logistic regression model was fitted using the glm function in R, resulting in coefficient estimates, odds ratios, and 95% confidence intervals. The statistical significance of each genetic element’s association with phenotypic resistance was set at *p* < 0.05.

## Results

### S. haemolyticus *is abundant and highly prevalent in stool samples of preterm infants hospitalised in the NICU*

**Figure 1.**
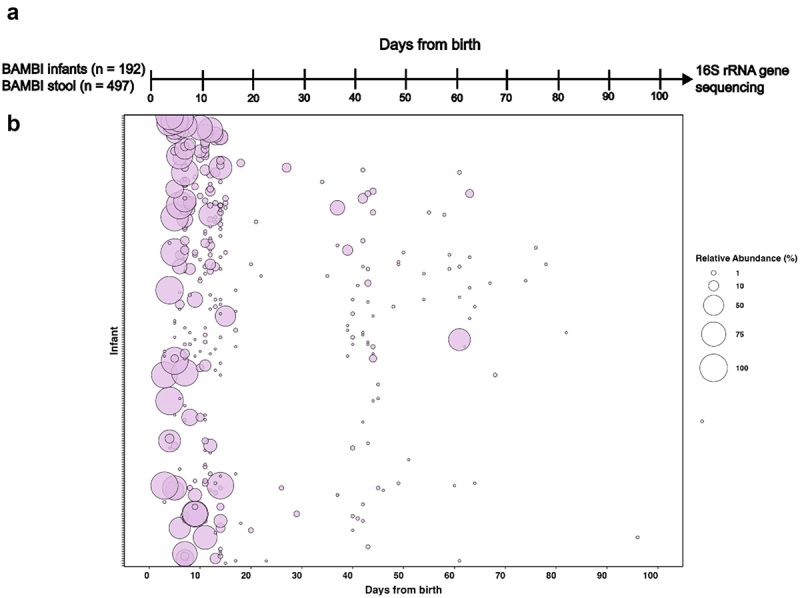
Estimation of *Staphylococcus haemolyticus* abundance in the preterm infant gut microbiota based on 16S rRNA gene sequencing data. The figure visualizes previously analyzed 16S rRNA gene sequencing data of 497 stool samples from 192 preterm infants.^19^ bubble plots show the relative abundance of OTUs identified as *Staphylococcus haemolyticus*.

To estimate the prevalence of *S. haemolyticus* in the preterm infant gut microbiota, we re-analyzed 16S rRNA gene sequencing data generated on stool samples (*n* = 497), from preterm infants (*n* = 192) enrolled in the Baby-Associated MicroBiota of the Intestine (BAMBI) study.^19^ In this dataset, OTUs that were identified as *S. haemolyticus* were abundant in the first 10 days after birth (Figure 1). While the abundance of *S. haemolyticus* decreased as the infants aged, *S. haemolyticus* was still detectable in some infants at later time points (30–70 days of age). Specifically, *S. haemolyticus* was detected in 22.2% of samples at time point 0–9 days, 29.5% of samples at 10–29 days, 11.7% of samples at 30–49 days, and 6.4% of samples at 50–99 days from birth.

### S. haemolyticus *isolates detected in majority of preterm infant stool samples belong to a clonal population*

**Figure 2.**
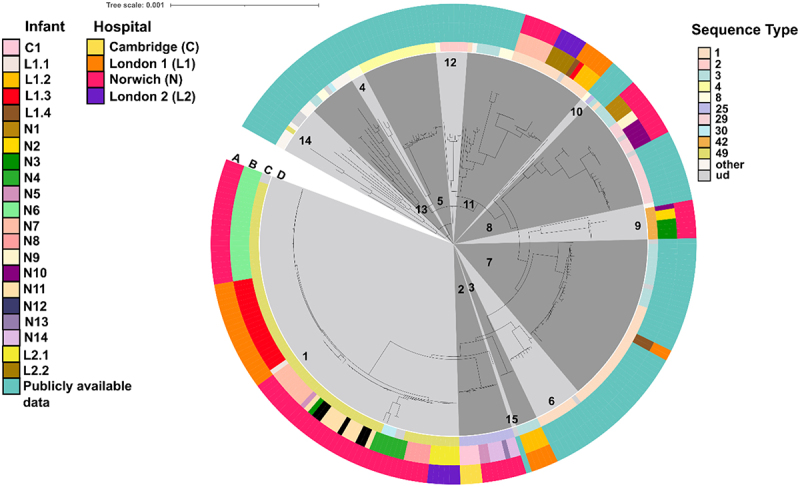
Midpoint-rooted phylogenetic tree illustrating core genome comparisons among strains sequenced in this study and publicly available strains (*n* = 266). The concentric rings display metadata related to stool isolates collected from preterm infants at various hospitals across the UK. Ring A indicates the hospital of origin for each isolate. Ring B denotes individual infants from whom the strains were isolated. Ring C shows multilocus sequence typing (MLST) designations for each strain. The central ring (ring D) uses alternating dark and light gray blocks to represent fastbaps clusters, numbered for reference. Sequence types (STs) with three or more isolates are labeled individually in the legend, while STs with fewer than three isolates are grouped under “other.” strains for which sequence types could not be determined are labeled as “ud” (undetermined).

To assess the genetic diversity of *S. haemolyticus* in the preterm infant gut, we isolated presumptive *S. haemolyticus* strains from stool specimens (*n* = 46), from preterm infants (*n* = 22) that were included in either the BAMBI or NeoM study (Figure S1). Whole genome sequencing confirmed the identity of 140 strains from 44 samples as *S. haemolyticus*, while the remaining 10 strains (from 2 samples) were identified as *Staphylococcus epidermidis*. The *S. haemolyticus*-positive stool samples originated from four hospitals in the East of England, specifically in Norwich (*n* = 28), two hospitals in London (London 1; *n* = 12; London 2; *n* = 3), and Cambridge (*n* = 1). All infants from which *S. haemolyticus* was isolated were treated in a NICU. A core genome phylogenetic tree, including 140 whole genome sequences from strains isolated here and 126 publicly available *S. haemolyticus* genomes, was constructed to study the relatedness of *S. haemolyticus* isolates (Figure 2). The most commonly found STs among the strains that were sequenced as part of this study were ST49 (78/140; 55.7%), ST1 (19/140 13.6%), and ST25 (12/140; 8.6%). Fastbaps analysis divided the 266 *S. haemolyticus* isolates into 22 clusters. The largest cluster, Cluster 1, consisted of 85/266 (32.0%) isolates, all coming from this study’s preterm infant cohort with isolates from three UK hospitals with 78/85 (91.7%) of these isolates belonging to ST49. Individual genomes in Cluster 1 differed between 0 and 369 core genome SNPs (cgSNPs) with an average cgSNP difference of 64.6 SNPs. Out of 916 instances in which cluster 1 strain pairs were closely related to each other (≤10 cgSNPs), 256 (27.9%) were found to be shared between different hospitals. If an infant was only colonized by Cluster 1 strains, the number of cgSNPs between strains isolated from the same stool sample ranged between 0 and 251 cgSNPs. Among the 44 stool samples from which *S. haemolyticus* was isolated, three (8.3%) returned strains from different clusters.

**Figure 3.**
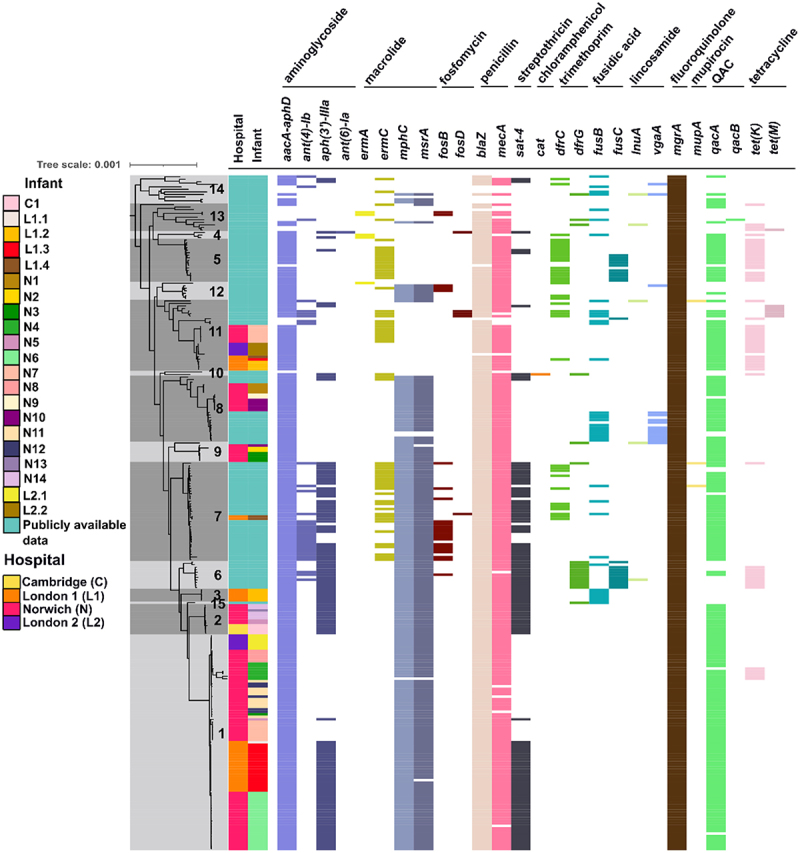
Antibiotic resistance gene (ARG) profiles in *Staphylococcus haemolyticus* isolates. This figure displays the presence or absence of specific ARGs and their associated antibiotic classes across isolates from preterm infants, grouped by their hospitals of origin. A core genome phylogenetic tree, with fastbaps clusters indicated, is shown on the left, together with information on the hospitals each infant was hospitalized in. Abbreviations: QAC – quaternary ammonium compounds.

In the *S. haemolyticus* genome sequences multiple antibiotic resistance genes (ARGs), conferring resistance to 13 antibiotic classes, were identified (Figure 3). The most commonly detected ARGs in our preterm infant cohort were the regulatory gene *mgrA* (140/140; 100%) the aminoglycoside resistance gene *aacA-aphD*, and the β-lactam resistance genes *blaZ* and *mecA* (all 139/140; 99.3%). The gene *qacA* (132/140; 94.3%), an efflux pump associated with tolerance toward disinfectants,^47^ was also nearly ubiquitous among the isolates from the preterm infant cohort.

**Figure 4.**
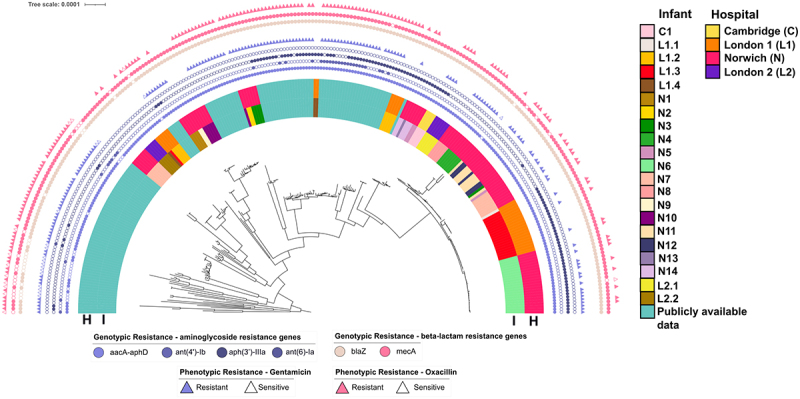
Presence of aminoglycoside and β-lactam resistance genes and outcome of antibiotic susceptibility testing. Coloured purple circles represent the presence of aminoglycoside resistance genes *aacA-aphD*, *ant(4’)-Ib*, *aph(3’)-IIIa*, and *ant(6)-iIa*. Coloured pink circles represent the presence of β-lactam resistance genes *blaZ* and *mecA*. Filled circles indicate that the gene is present, open circles indicate that the gene is absent. Filled triangles indicate phenotypic resistance to an antibiotic, open triangles indicate phenotypic sensitivity to antibiotics. The half-circle labeled “I” represents individual infants, and the half-circle labeled “H” indicates the hospitals from which these infants’ isolates were obtained, with the same color coding as in Figures 2 and 3. The midpoint-rooted phylogenetic tree is based on a core genome alignment of *S.* *haemolyticus* genomes.

We then performed antibiotic susceptibility testing on 58 *S. haemolyticus* strains which span the phylogenetic diversity of the strains isolated in this study. These data were then combined with antibiotic susceptibility data from 123 *S. haemolyticus* strains from a study by Cavanagh and colleagues^14^ to investigate the association between genotypic and phenotypic resistance to the antibiotics oxacillin (a β-lactam) and gentamicin (an aminoglycoside). Out of the 176 *S. haemolyticus* isolates tested for oxacillin resistance, 158 isolates harboring the *mecA* gene exhibited phenotypic resistance to oxacillin (158/176; 89.8%), 1 isolate harboring the *mecA* gene exhibited phenotypic sensitivity to oxacillin (1/176; 0.6%), 6 isolates lacking the *mecA* gene exhibited phenotypic resistance to oxacillin (6/176; 3.4%), while 11 isolates lacking the *mecA* gene exhibited phenotypic sensitivity to oxacillin (11/176; 6.3%). Analysis though an unconditional multivariable logistic regression model revealed a strong association between the presence of *mecA* and resistance to oxacillin (odds ratio [OR]: 158.00, 95% confidence interval [CI]: 134.63–183.92, *p* < 0.0001), in contrast to the presence of the other β-lactam resistance gene, *blaZ* (OR: 0.97, 95% CI: −0.78–1.22, *p* = 0.82) (Figure 4).

Of the 181 *S. haemolyticus* isolates tested for gentamicin resistance, 162 isolates encoding the *aacA-aphD* gene exhibited phenotypic resistance to gentamicin (162/181; 89.5%), whereas only 2 isolates encoding the *aacA-aphD* gene exhibited phenotypic sensitivity to gentamicin (2/181; 1.1%). Additionally, only 2 isolates lacking the *aacA-aphD* gene exhibited phenotypic resistance to gentamicin (2/181; 1.1%), while 15 isolates lacking the *aacA-aphD* gene exhibited phenotypic sensitivity to gentamicin (15/181; 8.3%). We found a significant positive association for the presence of *aacA-aphD* and phenotypic resistance to gentamicin (OR: 162.00, 95% CI: 138.31–188.24, *p* < 0.001), in contrast to the presence of other aminoglycoside genes (*ant(4’)-Ib* (OR = 0.18, 95% CI: 0.12–0.26, *p* < 0.001), *aph(3’)-IIIa* (OR = 0.46, 95% CI: 0.35–0.60; *p* < 0.001), and *ant(6)-Ia* (OR = 0.0061, 95% CI = −3.51 × 10^−4^ − 0.027; *p* < 0.001).

### *SCCmec elements are differentially distributed in* S. haemolyticus *isolates from the preterm infant cohort*

None of the isolates from our study had a complete SCC*mec* element. The distribution of SCC*mec* core elements *orfX* (*rlmH*), *mecA*, *mecR*, *mecI*, IS*431*, *ccrA*, *ccrB*, and *ccrC*^48^ was further investigated in our collection of *S. haemolyticus* isolates. Hierarchical clustering of observed SCC*mec* elements per isolate revealed distinct patterns of SCC*mec* components between isolates from our preterm infant cohort and publicly available data (Figure S2). A majority of *S. haemolyticus* isolates from our preterm infant BAMBI cohort (103/140; 73.6%) carried *orfX* (*rlmH*), *mecA*, and IS*431*. A smaller proportion of the isolates carried additional SCC*mec* elements, 19.3% (27/140) of isolates carried *orfX* (*rlmH*), *mecA*, IS*431*, and *ccrC* (27/140; 19.3%), 6.4% (9/140) of isolates had *orfX* (*rlmH*), *mecA*, IS*431, ccrA* and *ccrB* (9/140; 6.4%). Only a single isolate carried *orfX* (*rlmH*), *mecA*, IS*431, ccrA*, *ccrB* and *ccrC*. Utilising hybrid-assembled genomes, we examined the genetic context of the *mecA* regions within our *S. haemolyticus* preterm infant cohort and identified seven different genetic contexts (Figure S3).

### P*lasmid- and chromosome-mediated gentamicin resistance in* S. haemolyticus *isolates is encoded by a* *transposon, Tn*4001

**Figure 5.**
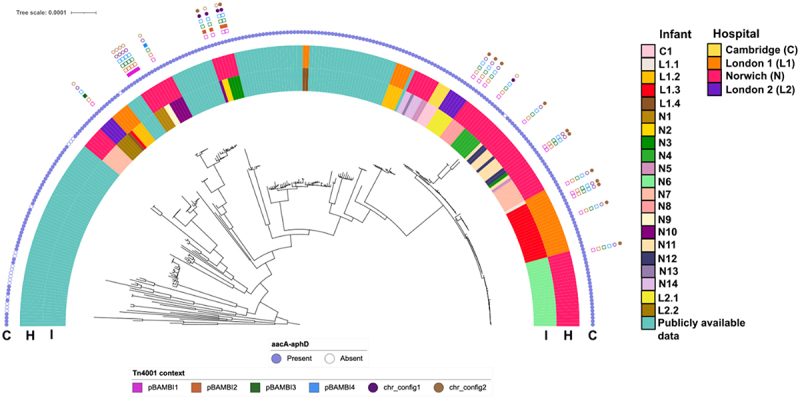
Genetic context of the Tn*4001* region found in *S.* *haemolyticus* isolates from the preterm infant cohort. Coloured purple circles represent the presence (filled circle) or absence (open circle) of *aacA-aphD* gene. Different genetic contexts of Tn*4001* are indicated: squares represent the configuration of Tn*4001* in plasmids (pBAMBI1 – pBAMBI4) and circles represent the configuration of Tn*4001* in the chromosome (chr1_config1 and chr_config 2) (Figure S3). The half-circle labeled “C” represents the Tn*4001* genetic context, the half-circle labeled “I” represents individual infants, and the half-circle labeled “H” indicates the hospitals from which these infants’ isolates were obtained and these use the same colors as in Figure 1 and 2. The midpoint-rooted phylogenetic tree is based on a core genome alignment of *S.* *haemolyticus* genomes.

We next examined the genetic context of gentamicin resistance gene *aacA-aphD* in *S. haemolyticus* isolates. In the genomes we generated for this study, this gentamicin resistance gene is always associated with a transposon, Tn*4001*, which has first been described in *S. aureus*.^49^ To conclusively map the genetic context of Tn4001, we generated hybrid assemblies for 22 strains and found that Tn*4001* was present on the chromosome in 12/22 (54.5%) of strains, and on a plasmid in 6/22 (27.3%) strains. In 4/22 (18.2%) strains Tn*4001* was present on both a plasmid and the chromosome (Figure 5).

The transposon was found in four distinct plasmids, which we have named pBAMBI1, pBAMBI2, pBAMBI3, and pBAMBI4 (Figure S4). In addition to Tn*4001*, all four of these plasmids also carry the *blaZRI* gene cluster, of which the first gene encodes an extracellular β-lactamase that is widespread in *Staphylococcus*, and the *qacRA* genes, which contribute to resistance to quaternary ammonium compounds and chlorhexidine.^50,51^ None of the plasmids were predicted to be conjugative, but three out of four plasmids (pBAMBI1, 2 and 4) were predicted by MOB-suite to be mobilizable, while pBAMBI3 was non-mobilizable (Table S3). All plasmids contained RepA_N replicons^52^ and were related to previously described plasmids in *S. epidermidis* and, in the case of pBAMBI2, which we found in three *S. haemolyticus* strains, to the backbone of the prototypical *S. aureus* multidrug resistance plasmid pSK1^53^ (Figure S4). We found two distinct configurations of the Tn*4001* chromosomal integration among the *S. haemolyticus* genomes (Figure S3), which we termed chr_configs 1 and 2 (Figure 5).

## Discussion

CoNS are currently understudied, even though they are a frequent cause of infection in neonates.^54^ After *S. epidermidis*, *S. haemolyticus* is among the CoNS most commonly causing bloodstream infections.^55^ Gut colonization by *S. haemolyticus* may be a risk factor for subsequent systemic infections in preterm infants.^56^ Prevalence of *S. haemolyticus* in the preterm infant gut is particularly high in the first few weeks post-partum.^57,58^ In this study, through a re-analysis of 16S rRNA gene data, we confirmed these observations, showing an initial high abundance of *S. haemolyticus* in the first ten days after birth, followed by a gradual decline as infants mature. We then employed high-throughput whole-genome sequencing to investigate the diversity of *S. haemolyticus* within the preterm infant gut. While our study has limitations, including the lack of access to detailed clinical data which precluded us to identify whether *S. haemolyticus* caused infections in these infants, and the relatively limited number of isolates from four sites in a geographically restricted region of England, our work has uncovered some relevant insights into the diversity of *S. haemolyticus*, including the evolution of multidrug-resistant clones.

The core genome phylogenetic analysis revealed a diverse population of *S. haemolyticus* among the isolates obtained from different UK hospitals. Among this population, Cluster 1 was most prominently represented among preterm infant isolates. These strains were identified in three different hospitals, demonstrating that Cluster 1 may represent a disseminated clonal population that has adapted to the gut colonization of the preterm infant gut, similar to what was proposed for a sub-population of *Staphylococcus capitis*.^59^ It is likely that transfer of patients or healthcare workers between hospitals, particularly between the two geographically close hospitals in London has led to the dissemination of Cluster 1 strains, similar to how methicillin-resistant *S. aureus* has spread among hospitals in the UK.^60–62^

Our whole genome sequence analysis identified the presence of multiple ARGs in *S. haemolyticus* from preterm infants, with particularly high prevalence of genes conferring resistance to β-lactams and aminoglycosides. The detection of these ARGs, particularly and *mecA* and *aacA-aphD* is relevant, as the antibiotics flucloxacillin and gentamicin are frequently the antibiotics of choice for babies with late-onset neonatal infection who are already in a neonatal unit,^10,63^ and there is thus a strong selective pressure for *S. haemolyticus* strains colonizing preterm neonates to acquire resistance to them. While our data strongly suggest that *mecA* and *aacA-aphD* are the most important resistance determinants for β-lactams and aminoglycosides in *S. haemolyticus*, we cannot rule out the presence of other genes and mutations that may contribute to resistance to these antibiotic classes in *S. haemolyticus*. Interestingly, using comparative genomics of nosocomial and commensal *S. haemolyticus* isolates, Pain and colleagues^64^ have proposed that the presence of both these genes (i.e. *aacA-aphD* and *mecA*) are primary indicators of hospital adaptation and pathogenicity. We found that the gene *aacA-aphD*, responsible for gentamicin resistance, is encoded within a transposon, Tn*4001*.^49^ This transposon was found in various genetic contexts, including on plasmids and in chromosomes, or in multiple copies distributed among both replicons, highlighting the potential for Tn*4001*’s mobility within *S. haemolyticus*. Notably, three plasmids were predicted to be mobilizable, suggesting that they may be transferred via conjugation if suitable conjugation machinery is provided *in trans*. Given the diversity of multiple staphylococcal species in the NICU environment, these plasmids may thus facilitate the dissemination of resistance genes across species boundaries. We note that these plasmids may also be selected for through the widespread use of disinfectants in hospital settings, due to the presence of the *qacA* gene, thus highlighting the potential for co-selection of antibiotic resistance and biocide tolerance in *S. haemolyticus*.^65^

In our dataset, ST49 was the dominant ST. Recent studies have found this ST to be common among healthy children in South Africa, but, interestingly, these strains did not carry *mecA*^66^. *mecA*^+^ ST49 strains have also been isolated from dogs in the USA^67^ and in animal veterinary practices in Switzerland,^68^ suggesting that this clone is globally disseminated and may be spreading between humans and animals. Other common STs (ST1 and ST25) in our dataset have previously been shown to be prevalent, multidrug-resistant lineages in clinical settings across the globe,^14,69,70^ suggesting that the species may contain multiple lineages of clinical concern. The near-ubiquitous spread of β-lactam and aminoglycoside resistance genes in *S. haemolyticus* isolates of preterm infants raises important questions about the selective pressures, and potentially the efficacy of antibiotic treatment of *S. haemolyticus* infections in preterm infants. Indeed, bloodstream infections caused by *S. haemolyticus* may be associated with higher morbidity in neonates, compared to the more commonly encountered species *S. epidermidis*.^71^ The combination of multidrug-resistance and virulence makes *S. haemolyticus* a particularly problematic CoNS in severely immunocompromised patient groups.^70,72–74 75^Future research could focus on generating a deeper understanding of the mechanisms by which *S. haemolyticus* can acquire, and potentially further disseminate, mobile genetic elements that carry antibiotic resistance genes.

## Supplementary Material

Supplemental Material

## Data Availability

Raw sequencing reads generated in this study are available at the European Nucleotide Archive (BioProject PRJNA1105567; https://www.ebi.ac.uk/ena/browser/view/PRJNA1105567).

## Preprint

An earlier version of this manuscript has been posted as a preprint on BioRxiv.[76]
