# Supplementary material for: Staphylococcus haemolyticus is a reservoir of antibiotic resistance genes in the preterm infant gut
Source: Gut Microbes. 2025 Jun 22;17(1):2519700. doi: 10.1080/19490976.2025.2519700 (PMC12323770; doi:10.1080/19490976.2025.2519700)
Supplement: Supplemental Material [file KGMI_A_2519700_SM3451.zip › TableS3_revised.docx]

**Table S3. Details of completely assembled *S. haemolyticus* plasmids**

| **Plasmid** | **Strain** | **Type** | **Predicted mobility** | **% GC** | **length (bp)** |
| --- | --- | --- | --- | --- | --- |
| pBAMBI1 | ARM6 | RepA_N: rep39 | mobilizable | 30.06 | 31610 |
| pBAMBI2 | ARM7 | RepA_N: repUS9 | mobilizable | 30.26 | 43415 |
| pBAMBI3 | ARM208 | Rep2: repUS46  RepA_N: repUS23 | non-mobilizable | 30.58 | 32980 |
| pBAMBI4 | ARM256 | RepA_N: rep39 | mobilizable | 30.39 | 42381 |
