# Supplementary material for: Staphylococcus haemolyticus is a reservoir of antibiotic resistance genes in the preterm infant gut
Source: Gut Microbes. 2025 Jun 22;17(1):2519700. doi: 10.1080/19490976.2025.2519700 (PMC12323770; doi:10.1080/19490976.2025.2519700)
Supplement: Supplemental Material [file KGMI_A_2519700_SM3451.zip › TableS1_15052025.docx]

**Table S1. Strains and metadata**

| isolate code | source_ID | infant_ID | location | sex | delivery | birth weight | gestational age (weeks, days) | fastbaps | ST | FastANI | OXA | GEN | accession number |
| --- | --- | --- | --- | --- | --- | --- | --- | --- | --- | --- | --- | --- | --- |
| Genomes sequenced as part of the current study | | | | | | | | | | | | | |
| ARM70 | C1.1 | C1 | Cambridge | M | C | 570 | 28,0 | 2 | 25 | 98.9 | NA | R | SRR30660753 |
| ARM71 | C1.1 | C1 | Cambridge | M | C | 570 | 28,0 | 2 | 25 | 98.8 | NA | R | SRR30660751 |
| ARM73 | C1.1 | C1 | Cambridge | M | C | 570 | 28,0 | 2 | 25 | 98.8 | NA | R | SRR30660750 |
| ARM74 | C1.1 | C1 | Cambridge | M | C | 570 | 28,0 | 2 | 25 | 98.6 | NA | R | SRR30887095 |
| ARM117 | L1.1.1 | L1.1 | London_1 | M | C | 1320 | 30,1 | 1 | 49 | 99 | NA | NA | SRR30620635 |
| ARM207 | L1.2.1 | L1.2 | London_1 | F | C | 1190 | 30,3 | 3 | 3 | 99.1 | R | NA | SRR30644698 |
| ARM205 | L1.2.1 | L1.2 | London_1 | F | C | 1190 | 30,3 | 3 | 3 | 99.1 | R | NA | SRR30644682 |
| ARM206 | L1.2.1 | L1.2 | London_1 | F | C | 1190 | 30,3 | 3 | 3 | 99.2 | NA | NA | SRR30644681 |
| ARM204 | L1.2.1 | L1.2 | London_1 | F | C | 1190 | 30,3 | 3 | 3 | 99.1 | R | NA | SRR30644683 |
| ARM203 | L1.2.1 | L1.2 | London_1 | F | C | 1190 | 30,3 | 3 | 3 | 99.1 | NA | NA | SRR30644684 |
| ARM125 | L1.2.2 | L1.2 | London_1 | F | V | 1190 | 30,3 | 11 | 1 | 98.8 | R | NA | SRR30620633 |
| ARM126 | L1.2.2 | L1.2 | London_1 | F | V | 1190 | 30,3 | 11 | 1 | 98.8 | R | NA | SRR30620632 |
| ARM123 | L1.2.2 | L1.2 | London_1 | F | V | 1190 | 30,3 | 11 | 1 | 98.8 | NA | NA | SRR30620634 |
| ARM127 | L1.2.2 | L1.2 | London_1 | F | V | 1190 | 30,3 | 11 | 1 | 98.8 | NA | NA | SRR30620631 |
| ARM100 | L1.3.1 | L1.3 | London_1 | M | C | 1370 | 30,3 | 1 | 49 | 99 | R | NA | SRR30620625 |
| ARM102 | L1.3.1 | L1.3 | London_1 | M | C | 1370 | 30,3 | 1 | 49 | 98.9 | NA | NA | SRR30620623 |
| ARM101 | L1.3.1 | L1.3 | London_1 | M | C | 1370 | 30,3 | 1 | 49 | 99.0 | NA | NA | SRR30620624 |
| ARM98 | L1.3.1 | L1.3 | London_1 | M | C | 1370 | 30,3 | 1 | 49 | 99.1 | NA | NA | SRR30887092 |
| ARM99 | L1.3.1 | L1.3 | London_1 | M | C | 1370 | 30,3 | 1 | 49 | 99.0 | NA | NA | SRR30887091 |
| ARM107 | L1.3.2 | L1.3 | London_1 | M | C | 1370 | 30,3 | 1 | 49 | 99.0 | NA | NA | SRR30620621 |
| ARM104 | L1.3.2 | L1.3 | London_1 | M | C | 1370 | 30,3 | 1 | 49 | 99.0 | NA | NA | SRR30620622 |
| ARM89 | L1.3.3 | L1.3 | London_1 | M | C | 1370 | 30,3 | 1 | 49 | 99.0 | NA | NA | SRR30887072 |
| ARM86 | L1.3.3 | L1.3 | London_1 | M | C | 1370 | 30,3 | 1 | 49 | 99.1 | NA | R | SRR30887083 |
| ARM88 | L1.3.3 | L1.3 | London_1 | M | C | 1370 | 30,3 | 1 | 49 | 99.0 | NA | NA | SRR30887073 |
| ARM87 | L1.3.3 | L1.3 | London_1 | M | C | 1370 | 30,3 | 1 | 49 | 99.0 | NA | R | SRR30887074 |
| ARM85 | L1.3.3 | L1.3 | London_1 | M | C | 1370 | 30,3 | 1 | 49 | 99.1 | NA | R | SRR30887094 |
| ARM91 | L1.3.4 | L1.3 | London_1 | M | C | 1370 | 30,3 | 1 | 49 | 99.0 | NA | NA | SRR30887070 |
| ARM94 | L1.3.4 | L1.3 | London_1 | M | C | 1370 | 30,3 | 1 | 49 | 99.0 | NA | NA | SRR30887093 |
| ARM90 | L1.3.4 | L1.3 | London_1 | M | C | 1370 | 30,3 | 1 | 49 | 99.1 | NA | NA | SRR30887071 |
| ARM92 | L1.3.4 | L1.3 | London_1 | M | C | 1370 | 30,3 | 1 | 49 | 99.0 | NA | NA | SRR30887069 |
| ARM93 | L1.3.4 | L1.3 | London_1 | M | C | 1370 | 30,3 | 1 | 49 | 99.0 | NA | NA | SRR30887068 |
| ARM265 | L1.3.5 | L1.3 | London_1 | M | C | 1370 | 30,3 | 1 | 49 | 99.1 | R | NA | SRR30660755 |
| ARM266 | L1.3.5 | L1.3 | London_1 | M | C | 1370 | 30,3 | 1 | 49 | 99.0 | NA | NA | SRR30660754 |
| ARM110 | L1.3.6 | L1.3 | London_1 | M | C | 1370 | 30,3 | 11 | 1 | 99.0 | NA | NA | SRR30620636 |
| CA-32 | L1.4.1 | L1.4 | London_1 | F | V | 1330 | 28,4 | 7 | 1 | 98.9 | NA | NA | SRR30183987 |
| CA-30 | L1.4.2 | L1.4 | London_1 | F | V | 1330 | 28,4 | 7 | 1 | 98.8 | NA | NA | SRR30183988 |
| CA-29 | L1.4.3 | L1.4 | London_1 | F | V | 1330 | 28,4 | 11 | 1 | 98.8 | NA | NA | SRR30183989 |
| ARM247 | L2.1.1 | L2.1 | London_2 | M | V | 510 | 23,1 | 1 | 49 | 99.1 | R | NA | SRR30660743 |
| ARM248 | L2.1.1 | L2.1 | London_2 | M | V | 510 | 23,1 | 1 | 49 | 99.0 | NA | NA | SRR30660762 |
| ARM225 | L2.1.2 | L2.1 | London_2 | M | V | 510 | 23,1 | 1 | 49 | 99.0 | NA | NA | SRR30644691 |
| ARM224 | L2.1.2 | L2.1 | London_2 | M | V | 510 | 23,1 | 1 | 49 | 99.0 | NA | NA | SRR30644692 |
| ARM227 | L2.1.2 | L2.1 | London_2 | M | V | 510 | 23,1 | 1 | 49 | 98.9 | NA | NA | SRR30644690 |
| ARM228 | L2.1.2 | L2.1 | London_2 | M | V | 510 | 23,1 | 1 | 49 | 98.9 | NA | NA | SRR30644689 |
| ARM212 | L2.2.1 | L2.2 | London_2 | F | V | 850 | 25,3 | 11 | 1 | 98.8 | NA | NA | SRR30644693 |
| ARM209 | L2.2.1 | L2.2 | London_2 | F | V | 850 | 25,3 | 11 | NA | 98.8 | NA | NA | SRR30644696 |
| ARM208 | L2.2.1 | L2.2 | London_2 | F | V | 850 | 25,3 | 11 | 1 | 98.8 | R | NA | SRR30644697 |
| ARM210 | L2.2.1 | L2.2 | London_2 | F | V | 850 | 25,3 | 11 | 1 | 98.8 | R | NA | SRR30644695 |
| ARM211 | L2.2.1 | L2.2 | London_2 | F | V | 850 | 25,3 | 11 | 1 | 98.8 | NA | NA | SRR30644694 |
| ARM4 | N1.1 | N1 | Norwich | F | V | 1384 | 25,4 | 8 | 3 | 98.7 | NA | R | SRR30146286 |
| ARM2 | N1.1 | N1 | Norwich | F | V | 1384 | 25,4 | 8 | 3 | 98.7 | R | R | SRR30146296 |
| ARM6 | N1.1 | N1 | Norwich | F | V | 1384 | 25,4 | 8 | 3 | 98.6 | NA | R | SRR30146294 |
| ARM1 | N1.1 | N1 | Norwich | F | V | 1384 | 25,4 | 8 | 3 | 98.7 | R | R | SRR30146297 |
| ARM257 | N10.1 | N10 | Norwich | M | C | 1544 | 30 | 8 | 29 | 98.8 | NA | NA | SRR30660758 |
| ARM254 | N10.1 | N10 | Norwich | M | C | 1544 | 30 | 8 | 29 | 98.8 | NA | NA | SRR30660761 |
| ARM256 | N10.1 | N10 | Norwich | M | C | 1544 | 30 | 8 | 29 | 98.7 | NA | NA | SRR30660759 |
| ARM258 | N10.1 | N10 | Norwich | M | C | 1544 | 30 | 8 | NA | 98.8 | NA | NA | SRR30660757 |
| ARM255 | N10.1 | N10 | Norwich | M | C | 1544 | 30 | 8 | 29 | 98.8 | NA | NA | SRR30660760 |
| ARM262 | N10.2 | N10 | Norwich | M | C | 1544 | 30 | 9 | 42 | 99.0 | NA | NA | SRR30660756 |
| ARM62 | N11.2 | N11 | Norwich | M | V | 831 | 25,5 | 1 | 49 | 99.0 | R | R | SRR30146292 |
| P80B-1 | N11.1 | N11 | Norwich | M | V | 831 | 25,5 | 1 | 49 | 99.0 | NA | NA | SRR30887081 |
| P80B-2 | N11.1 | N11 | Norwich | M | V | 831 | 25,5 | 1 | 49 | 99.0 | NA | NA | SRR30887080 |
| ARM56 | N11.1 | N11 | Norwich | M | V | 831 | 25,5 | 1 | 49 | 99.0 | R | R | SRR30146291 |
| ARM58 | N11.1 | N11 | Norwich | M | V | 831 | 25,5 | 1 | 49 | 99.0 | R | R | SRR30146284 |
| ARM57 | N11.1 | N11 | Norwich | M | V | 831 | 25,5 | 1 | 49 | 98.9 | R | R | SRR30146285 |
| ARM59 | N11.1 | N11 | Norwich | M | V | 831 | 25,5 | 1 | 49 | 99.1 | R | R | SRR30146295 |
| ARM55 | N11.1 | N11 | Norwich | M | V | 831 | 25,5 | 1 | 49 | 99.0 | R | R | SRR28887083 |
| ARM61 | N11.2 | N11 | Norwich | M | V | 831 | 25,5 | 1 | 49 | 99.0 | R | R | SRR30146293 |
| P86J-1 | N12.1 | N12 | Norwich | M | C | 1400 | 31 | 1 | 49 | 99.0 | NA | NA | SRR30887079 |
| P86J-2 | N12.1 | N12 | Norwich | M | C | 1400 | 31 | 1 | 49 | 99.0 | NA | NA | SRR30887078 |
| P86J-3 | N12.1 | N12 | Norwich | M | C | 1400 | 31 | 1 | 49 | 99.0 | NA | NA | SRR30887077 |
| P86J-4 | N12.1 | N12 | Norwich | M | C | 1400 | 31 | 1 | 49 | 99.0 | NA | NA | SRR30887076 |
| P86J-5 | N12.1 | N12 | Norwich | M | C | 1400 | 31 | 1 | 49 | 99.1 | NA | NA | SRR30887075 |
| ARM132 | N13.1 | N13 | Norwich | F | C | 700 | 25,0 | 2 | 25 | 98.8 | NA | NA | SRR30620630 |
| ARM134 | N14.1 | N14 | Norwich | M | C | 1443 | 31,4 | 2 | 25 | 98.9 | NA | NA | SRR30644700 |
| ARM135 | N14.1 | N14 | Norwich | M | C | 1443 | 31,4 | 2 | 25 | 98.9 | NA | NA | SRR30644699 |
| ARM136 | N14.1 | N14 | Norwich | M | C | 1443 | 31,4 | 2 | 25 | 98.9 | NA | NA | SRR30644688 |
| ARM137 | N14.1 | N14 | Norwich | M | C | 1443 | 31,4 | 2 | 25 | 98.9 | NA | NA | SRR30644687 |
| ARM133 | N14.1 | N14 | Norwich | M | C | 1443 | 31,4 | 2 | 25 | 98.9 | R | NA | SRR30620629 |
| ARM7 | N2.1 | N2 | Norwich | M | V | 1700 | 30,1 | 9 | 42 | 99.1 | R | R | SRR30183990 |
| ARM9 | N2.1 | N2 | Norwich | M | V | 1700 | 30,1 | 9 | 42 | 99.0 | R | R | SRR30183999 |
| ARM23 | N3.1 | N3 | Norwich | M | V | 1374 | 30 | 1 | 49 | 99.0 | NA | R | SRR28824051 |
| ARM25 | N3.1 | N3 | Norwich | M | V | 1374 | 30 | 9 | 42 | 99.1 | R | R | SRR30146289 |
| ARM27 | N3.1 | N3 | Norwich | M | V | 1374 | 30 | 9 | 42 | 99.1 | R | R | SRR30146287 |
| ARM26 | N3.1 | N3 | Norwich | M | V | 1374 | 30 | 9 | 42 | 99.1 | R | R | SRR30146288 |
| ARM24 | N3.1 | N3 | Norwich | M | V | 1374 | 30 | 9 | 42 | 99.1 | R | R | SRR30146290 |
| ARM34 | N4.1 | N4 | Norwich | F | V | 1477 | 30 | 1 | NA | 99.0 | NA | R | SRR28824050 |
| ARM36 | N4.1 | N4 | Norwich | F | V | 1477 | 30 | 1 | NA | 98.9 | NA | R | SRR28824048 |
| ARM38 | N4.1 | N4 | Norwich | F | V | 1477 | 30 | 1 | 30 | 98.9 | NA | R | SRR28824047 |
| ARM39 | N4.1 | N4 | Norwich | F | V | 1477 | 30 | 1 | 30 | 99.0 | NA | R | SRR28824046 |
| ARM35 | N4.1 | N4 | Norwich | F | V | 1477 | 30 | 1 | 49 | 99.0 | NA | R | SRR28824049 |
| ARM192 | N4.2 | N4 | Norwich | F | V | 1477 | 30 | 1 | 49 | 98.9 | NA | S | SRR30644685 |
| ARM188 | N4.2 | N4 | Norwich | F | V | 1477 | 30 | 1 | 30 | 98.9 | NA | NA | SRR30644686 |
| P73D-2 | N5.1 | N5 | Norwich | F | C | 1504 | 30 | 1 | 49 | 98.9 | NA | NA | SRR30887088 |
| P73C-5 | N5.1 | N5 | Norwich | F | C | 1504 | 30 | 2 | 25 | 98.8 | NA | NA | SRR30887089 |
| P73C-4 | N5.1 | N5 | Norwich | F | C | 1504 | 30 | 2 | 25 | 98.9 | NA | NA | SRR30887090 |
| ARM239 | N6.1 | N6 | Norwich | M | C | 1382 | 31 | 1 | 49 | 99.1 | R | R | SRR30660748 |
| ARM42 | N6.1 | N6 | Norwich | M | C | 1382 | 31 | 1 | 49 | 99.0 | NA | R | SRR28824043 |
| ARM240 | N6.1 | N6 | Norwich | M | C | 1382 | 31 | 1 | 49 | 99.1 | R | R | SRR30660747 |
| ARM41 | N6.1 | N6 | Norwich | M | C | 1382 | 31 | 1 | 49 | 99.1 | NA | R | SRR28824044 |
| ARM40 | N6.1 | N6 | Norwich | M | C | 1382 | 31 | 1 | NA | 99.0 | NA | R | SRR28824045 |
| ARM43 | N6.1 | N6 | Norwich | M | C | 1382 | 31 | 1 | NA | 99.0 | NA | R | SRR28824042 |
| ARM44 | N6.1 | N6 | Norwich | M | C | 1382 | 31 | 1 | 49 | 99.0 | NA | R | SRR28887086 |
| ARM243 | N6.1 | N6 | Norwich | M | C | 1382 | 31 | 1 | 49 | 99.1 | R | R | SRR30660744 |
| ARM242 | N6.1 | N6 | Norwich | M | C | 1382 | 31 | 1 | 49 | 99.1 | R | R | SRR30660745 |
| ARM241 | N6.1 | N6 | Norwich | M | C | 1382 | 31 | 1 | 49 | 99.1 | R | NA | SRR30660746 |
| CA-93 | N6.2 | N6 | Norwich | M | C | 1382 | 31 | 1 | 49 | 99.1 | R | R | SRR30620628 |
| CA-95 | N6.3 | N6 | Norwich | M | C | 1382 | 31 | 1 | 49 | 99.1 | NA | NA | SRR30620626 |
| ARM49 | N6.4 | N6 | Norwich | M | C | 1382 | 31 | 1 | 49 | 99.1 | S | R | SRR28887079 |
| ARM45 | N6.4 | N6 | Norwich | M | C | 1382 | 31 | 1 | 49 | 99.0 | NA | R | SRR28887085 |
| ARM48 | N6.4 | N6 | Norwich | M | C | 1382 | 31 | 1 | 49 | 99.1 | NA | R | SRR28887080 |
| ARM234 | N6.4 | N6 | Norwich | M | C | 1382 | 31 | 1 | 49 | 99.1 | R | R | SRR30660764 |
| ARM47 | N6.4 | N6 | Norwich | M | C | 1382 | 31 | 1 | 49 | 99.1 | NA | R | SRR28887081 |
| ARM235 | N6.4 | N6 | Norwich | M | C | 1382 | 31 | 1 | 49 | 99.0 | NA | NA | SRR30660763 |
| ARM236 | N6.4 | N6 | Norwich | M | C | 1382 | 31 | 1 | 49 | 99.0 | NA | NA | SRR30660752 |
| ARM46 | N6.4 | N6 | Norwich | M | C | 1382 | 31 | 1 | 49 | 99.1 | R | R | SRR28887082 |
| ARM238 | N6.4 | N6 | Norwich | M | C | 1382 | 31 | 1 | 49 | 99.0 | NA | NA | SRR30660749 |
| CA-61 | N6.5 | N6 | Norwich | M | C | 1382 | 31 | 1 | 49 | 99.0 | NA | NA | SRR30183995 |
| CA-94 | N6.6 | N6 | Norwich | M | C | 1382 | 31 | 1 | 49 | 99.1 | NA | NA | SRR30620627 |
| P75CTR | N7.1 | N7 | Norwich | M | V | 1262 | 27 | 1 | 49 | 99.1 | NA | NA | SRR30887082 |
| ARM50 | N7.2 | N7 | Norwich | M | V | 1262 | 27 | 1 | 49 | 99.1 | R | R | SRR28887078 |
| ARM53 | N7.2 | N7 | Norwich | M | V | 1262 | 27 | 1 | 49 | 99.1 | R | R | SRR28887075 |
| ARM52 | N7.2 | N7 | Norwich | M | V | 1262 | 27 | 1 | 49 | 99.1 | R | R | SRR28887076 |
| ARM54 | N7.2 | N7 | Norwich | M | V | 1262 | 27 | 1 | 49 | 99.1 | R | R | SRR28887084 |
| ARM51 | N7.2 | N7 | Norwich | M | V | 1262 | 27 | 1 | 49 | 99.0 | R | R | SRR28887077 |
| P75B-2 | N7.2 | N7 | Norwich | M | V | 1262 | 27 | 11 | 1 | 98.9 | NA | NA | SRR30887086 |
| P75B-5 | N7.2 | N7 | Norwich | M | V | 1262 | 27 | 11 | 1 | 98.9 | NA | NA | SRR30887084 |
| P75B-1 | N7.2 | N7 | Norwich | M | V | 1262 | 27 | 11 | 1 | 98.9 | NA | NA | SRR30887087 |
| P75B-3 | N7.2 | N7 | Norwich | M | V | 1262 | 27 | 11 | 1 | 98.9 | NA | NA | SRR30887085 |
| CA-63 | N7.3 | N7 | Norwich | M | V | 1262 | 27 | 1 | 49 | 99.1 | NA | NA | SRR30183994 |
| CA-58 | N7.4 | N7 | Norwich | M | V | 1262 | 27 | 1 | 49 | 99.1 | R | R | SRR30183997 |
| CA-59 | N7.5 | N7 | Norwich | M | V | 1262 | 27 | 11 | 1 | 98.9 | NA | NA | SRR30183996 |
| CA-64 | N7.6 | N7 | Norwich | M | V | 1262 | 27 | 11 | 1 | 98.9 | NA | NA | SRR30620638 |
| CA-65 | N7.2 | N7 | Norwich | M | V | 1262 | 27 | 11 | 1 | 98.8 | NA | NA | SRR30620637 |
| ARM69 | N8.1 | N8 | Norwich | M | V | 1409 | 27 | 1 | 49 | 99.0 | R | R | SRR30183991 |
| ARM68 | N8.1 | N8 | Norwich | M | V | 1409 | 27 | 1 | 49 | 99.0 | R | R | SRR30183992 |
| ARM65 | N8.1 | N8 | Norwich | M | V | 1409 | 27 | 1 | 49 | 99.0 | R | R | SRR30184002 |
| ARM67 | N8.1 | N8 | Norwich | M | V | 1409 | 27 | 1 | 49 | 99.0 | R | R | SRR30183993 |
| ARM66 | N8.1 | N8 | Norwich | M | V | 1409 | 27 | 1 | 49 | 99.0 | R | R | SRR30184001 |
| CA-48 | N9.1 | N9 | Norwich | F | C | 1110 | 30 | 8 | 29 | 98.7 | NA | NA | SRR30184000 |
| CA-49 | N9.2 | N9 | Norwich | F | C | 1110 | 30 | 8 | 29 | 98.8 | NA | NA | SRR30183998 |
|  |  |  |  |  |  | | | | |  |  |  |  |
| Publicly available genome sequences included in this study | | | | | | | | | | | | | |
| isolate code | **sourceID** | **infantID** | **geographicloc** | **sex** | **delivery** | **birthweight** | | **fastbaps** | **ST** |  | **pheno_oxa** | **pheno_gen** | **accession** |
| CUGJ01 | ncbi | NA | NA | NA | NA | NA |  | NA | 1 | 98.7 | R | R | ERR085272 |
| CUHJ01 | ncbi | NA | NA | NA | NA | NA |  | NA | 1 | 98.7 | R | R | ERR085292 |
| CUGV01 | ncbi | NA | NA | NA | NA | NA |  | NA | 1 | 98.7 | R | R | ERR085282 |
| CUGX01 | ncbi | NA | NA | NA | NA | NA |  | NA | 1 | 98.7 | R | R | ERR085285 |
| CUGK01 | ncbi | NA | NA | NA | NA | NA |  | NA | 1 | 98.8 | R | R | ERR085273 |
| CUGL01 | ncbi | NA | NA | NA | NA | NA |  | NA | 1 | 98.8 | R | R | ERR085274 |
| CUHC01 | ncbi | NA | NA | NA | NA | NA |  | NA | 1 | 98.8 | R | R | ERR085237 |
| CUCT01 | ncbi | NA | NA | NA | NA | NA |  | NA | 1 | 98.8 | R | R | ERR085176 |
| CUCR01 | ncbi | NA | NA | NA | NA | NA |  | NA | NA | 98.9 | R | R | ERR085175 |
| CUCX01 | ncbi | NA | NA | NA | NA | NA |  | NA | 1 | 98.8 | R | R | ERR085181 |
| CUFD01 | ncbi | NA | NA | NA | NA | NA |  | NA | 1 | 98.8 | R | R | ERR085241 |
| CUFE01 | ncbi | NA | NA | NA | NA | NA |  | NA | 1 | 98.8 | R | R | ERR085242 |
| CUGH01 | ncbi | NA | NA | NA | NA | NA |  | NA | 1 | 98.8 | R | R | ERR085270 |
| CUGT01 | ncbi | NA | NA | NA | NA | NA |  | NA | 1 | 98.8 | R | R | ERR085236 |
| CUHP01 | ncbi | NA | NA | NA | NA | NA |  | NA | 1 | 98.5 | R | R | ERR085240 |
| CUHM01 | ncbi | NA | NA | NA | NA | NA |  | NA | 1 | 98.7 | R | R | ERR085238 |
| CUGR01 | ncbi | NA | NA | NA | NA | NA |  | NA | 1 | 98.7 | R | R | ERR085280 |
| CUHL01 | ncbi | NA | NA | NA | NA | NA |  | NA | 1 | 98.7 | R | R | ERR085298 |
| CUFC01 | ncbi | NA | NA | NA | NA | NA |  | NA | 1 | 98.9 | NA | R | ERR085232 |
| CUGZ01 | ncbi | NA | NA | NA | NA | NA |  | NA | 1 | 98.9 | R | R | ERR085287 |
| CUDN01 | ncbi | NA | NA | NA | NA | NA |  | NA | 1 | 98.8 | R | R | ERR085193 |
| CUEU01 | ncbi | NA | NA | NA | NA | NA |  | NA | 1 | 98.8 | R | R | ERR085223 |
| CUGO01 | ncbi | NA | NA | NA | NA | NA |  | NA | 1 | 98.7 | R | R | ERR085277 |
| CUFB01 | ncbi | NA | NA | NA | NA | NA |  | NA | 3 | 98.6 | R | R | ERR085230 |
| CUDB01 | ncbi | NA | NA | NA | NA | NA |  | NA | 3 | 98.7 | R | R | ERR085182 |
| CUCZ01 | ncbi | NA | NA | NA | NA | NA |  | NA | 3 | 98.7 | R | R | ERR085183 |
| CUDQ01 | ncbi | NA | NA | NA | NA | NA |  | NA | 3 | 98.8 | R | R | ERR085197 |
| CUCU01 | ncbi | NA | NA | NA | NA | NA |  | NA | 3 | 98.8 | R | R | ERR085177 |
| CUDM01 | ncbi | NA | NA | NA | NA | NA |  | NA | 3 | 98.8 | R | R | ERR085190 |
| CUDE01 | ncbi | NA | NA | NA | NA | NA |  | NA | 3 | 98.5 | R | R | ERR085186 |
| CUDJ01 | ncbi | NA | NA | NA | NA | NA |  | NA | 3 | 98.8 | R | R | ERR085191 |
| CUFX01 | ncbi | NA | NA | NA | NA | NA |  | NA | 3 | 98.7 | R | R | ERR085262 |
| CUEY01 | ncbi | NA | NA | NA | NA | NA |  | NA | 3 | 98.7 | R | R | ERR085228 |
| CUDC01 | ncbi | NA | NA | NA | NA | NA |  | NA | 3 | 98.8 | R | R | ERR085184 |
| CUGG01 | ncbi | NA | NA | NA | NA | NA |  | NA | 3 | 98.9 | R | R | ERR085269 |
| CUGD01 | ncbi | NA | NA | NA | NA | NA |  | NA | 3 | 98.8 | R | R | ERR085267 |
| CUGU01 | ncbi | NA | NA | NA | NA | NA |  | NA | 3 | 98.8 | R | R | ERR085283 |
| CUCV01 | ncbi | NA | NA | NA | NA | NA |  | NA | 3 | 98.8 | R | R | ERR085178 |
| CUEW01 | ncbi | NA | NA | NA | NA | NA |  | NA | NA | 98.8 | R | R | ERR085225 |
| CUHQ01 | ncbi | NA | NA | NA | NA | NA |  | NA | 1 | 98.8 | R | R | ERR085239 |
| CUGW01 | ncbi | NA | NA | NA | NA | NA |  | NA | 1 | 98.8 | R | NA | ERR085284 |
| CUGF01 | ncbi | NA | NA | NA | NA | NA |  | NA | 1 | 98.9 | R | R | ERR085235 |
| CUCY01 | ncbi | NA | NA | NA | NA | NA |  | NA | 1 | 98.8 | R | R | ERR085180 |
| CUFM01 | ncbi | NA | NA | NA | NA | NA |  | NA | 1 | 98.8 | R | R | ERR085233 |
| CUEM01 | ncbi | NA | NA | NA | NA | NA |  | NA | 1 | 98.8 | R | R | ERR085217 |
| CUFV01 | ncbi | NA | NA | NA | NA | NA |  | NA | 1 | 98.9 | R | R | ERR085234 |
| CUFF01 | ncbi | NA | NA | NA | NA | NA |  | NA | 1 | 98.8 | R | R | ERR085243 |
| CUEN01 | ncbi | NA | NA | NA | NA | NA |  | NA | 1 | 98.8 | R | R | ERR085218 |
| CUGQ01 | ncbi | NA | NA | NA | NA | NA |  | NA | 1 | 98.8 | R | R | ERR085279 |
| CUET01 | ncbi | NA | NA | NA | NA | NA |  | NA | NA | 98.8 | R | R | ERR085224 |
| CUDI01 | ncbi | NA | NA | NA | NA | NA |  | NA | NA | 99.0 | R | R | ERR085189 |
| CUDX01 | ncbi | NA | NA | NA | NA | NA |  | NA | 58 | 98.7 | R | R | ERR085205 |
| CUED01 | ncbi | NA | NA | NA | NA | NA |  | NA | NA | 98.5 | S | S | ERR085210 |
| CUHI01 | ncbi | NA | NA | NA | NA | NA |  | NA | 25 | 98.7 | R | R | ERR085296 |
| CUFZ01 | ncbi | NA | NA | NA | NA | NA |  | NA | 4 | 98.6 | R | R | ERR085263 |
| CUHH01 | ncbi | NA | NA | NA | NA | NA |  | NA | 4 | 98.7 | R | R | ERR085293 |
| CUFR01 | ncbi | NA | NA | NA | NA | NA |  | NA | 4 | 98.7 | R | R | ERR085257 |
| CUGB01 | ncbi | NA | NA | NA | NA | NA |  | NA | 4 | 98.7 | R | R | ERR085265 |
| CUHD01 | ncbi | NA | NA | NA | NA | NA |  | NA | 4 | 98.7 | R | R | ERR085290 |
| CUDP01 | ncbi | NA | NA | NA | NA | NA |  | NA | 4 | 98.6 | R | R | ERR085195 |
| CUHK01 | ncbi | NA | NA | NA | NA | NA |  | NA | 4 | 98.6 | R | S | ERR085297 |
| CUHA01 | ncbi | NA | NA | NA | NA | NA |  | NA | 4 | 98.5 | R | R | ERR085289 |
| CUDD01 | ncbi | NA | NA | NA | NA | NA |  | NA | 4 | 98.5 | R | R | ERR085185 |
| CUDR01 | ncbi | NA | NA | NA | NA | NA |  | NA | 4 | 98.6 | R | R | ERR085196 |
| CUDF01 | ncbi | NA | NA | NA | NA | NA |  | NA | 4 | 98.5 | R | R | ERR085187 |
| CUCW01 | ncbi | NA | NA | NA | NA | NA |  | NA | 4 | 98.6 | R | R | ERR085179 |
| CUEH01 | ncbi | NA | NA | NA | NA | NA |  | NA | 4 | 98.6 | R | R | ERR085212 |
| CUDZ01 | ncbi | NA | NA | NA | NA | NA |  | NA | 4 | 98.6 | R | R | ERR085203 |
| CUDS01 | ncbi | NA | NA | NA | NA | NA |  | NA | 4 | 98.7 | R | R | ERR085198 |
| CUDU01 | ncbi | NA | NA | NA | NA | NA |  | NA | 4 | 98.7 | R | R | ERR085201 |
| CUDT01 | ncbi | NA | NA | NA | NA | NA |  | NA | 4 | 98.6 | NA | R | ERR085199 |
| CUCQ01 | ncbi | NA | NA | NA | NA | NA |  | NA | 29 | 98.5 | R | R | ERR085172 |
| CUCM01 | ncbi | NA | NA | NA | NA | NA |  | NA | 29 | 98.6 | R | R | ERR085169 |
| CUCS01 | ncbi | NA | NA | NA | NA | NA |  | NA | 29 | 98.6 | R | R | ERR085173 |
| CUCO01 | ncbi | NA | NA | NA | NA | NA |  | NA | 29 | 98.7 | R | R | ERR085174 |
| CUDL01 | ncbi | NA | NA | NA | NA | NA |  | NA | 29 | 98.7 | R | R | ERR085192 |
| CUCK01 | ncbi | NA | NA | NA | NA | NA |  | NA | 29 | 98.6 | R | R | ERR085167 |
| CUCP01 | ncbi | NA | NA | NA | NA | NA |  | NA | 29 | 98.5 | R | R | ERR085170 |
| CUCJ01 | ncbi | NA | NA | NA | NA | NA |  | NA | 29 | 98.5 | R | R | ERR085166 |
| CUEL01 | ncbi | NA | NA | NA | NA | NA |  | NA | 29 | 98.6 | R | R | ERR085216 |
| CUCI01 | ncbi | NA | NA | NA | NA | NA |  | NA | 29 | 98.6 | R | R | ERR085165 |
| CUEK01 | ncbi | NA | NA | NA | NA | NA |  | NA | 29 | 98.6 | R | R | ERR085215 |
| CUDO01 | ncbi | NA | NA | NA | NA | NA |  | NA | 3 | 98.9 | R | R | ERR085194 |
| CUCN01 | ncbi | NA | NA | NA | NA | NA |  | NA | NA | 98.6 | R | R | ERR085171 |
| CUDW01 | ncbi | NA | NA | NA | NA | NA |  | NA | 2 | 98.9 | R | R | ERR085202 |
| CUDY01 | ncbi | NA | NA | NA | NA | NA |  | NA | 2 | 98.9 | R | R | ERR085204 |
| CUEJ01 | ncbi | NA | NA | NA | NA | NA |  | NA | 2 | 99.0 | R | R | ERR085214 |
| CUFJ01 | ncbi | NA | NA | NA | NA | NA |  | NA | 2 | 98.7 | R | R | ERR085249 |
| CUFP01 | ncbi | NA | NA | NA | NA | NA |  | NA | 8 | 98.8 | R | R | ERR085256 |
| CUFN01 | ncbi | NA | NA | NA | NA | NA |  | NA | 2 | 98.8 | R | R | ERR085251 |
| CUGM01 | ncbi | NA | NA | NA | NA | NA |  | NA | 3 | 98.7 | R | R | ERR085276 |
| CUGY01 | ncbi | NA | NA | NA | NA | NA |  | NA | 3 | 98.8 | R | R | ERR085286 |
| CUFY01 | ncbi | NA | NA | NA | NA | NA |  | NA | 3 | 98.6 | R | R | ERR085260 |
| CUEB01 | ncbi | NA | NA | NA | NA | NA |  | NA | 3 | 98.6 | R | R | ERR085206 |
| CUFK01 | ncbi | NA | NA | NA | NA | NA |  | NA | 3 | 98.7 | R | R | ERR085246 |
| CUFG01 | ncbi | NA | NA | NA | NA | NA |  | NA | 8 | 98.6 | R | R | ERR085244 |
| CVRV01 | ncbi | NA | NA | NA | NA | NA |  | NA | 8 | 98.5 | NA | NA | ERR085258 |
| CUFA01 | ncbi | NA | NA | NA | NA | NA |  | NA | 8 | 98.6 | S | S | ERR085231 |
| CUEV01 | ncbi | NA | NA | NA | NA | NA |  | NA | 8 | 98.5 | S | S | ERR085226 |
| CUHE01 | ncbi | NA | NA | NA | NA | NA |  | NA | 1 | 98.8 | R | R | ERR085294 |
| CUFI01 | ncbi | NA | NA | NA | NA | NA |  | NA | 9 | 98.3 | R | S | ERR085245 |
| CUFH01 | ncbi | NA | NA | NA | NA | NA |  | NA | 59 | 98.4 | R | S | ERR085247 |
| CUHB01 | ncbi | NA | NA | NA | NA | NA |  | NA | 58 | 98.4 | R | R | ERR085288 |
| CUEX01 | ncbi | NA | NA | NA | NA | NA |  | NA | 9 | 98.2 | R | R | ERR085227 |
| CUHG01 | ncbi | NA | NA | NA | NA | NA |  | NA | 30 | 98.5 | R | S | ERR085295 |
| CUEI01 | ncbi | NA | NA | NA | NA | NA |  | NA | NA | 98.4 | S | S | ERR085211 |
| CUFW01 | ncbi | NA | NA | NA | NA | NA |  | NA | 3 | 98.5 | R | S | ERR085261 |
| CUEF01 | ncbi | NA | NA | NA | NA | NA |  | NA | 3 | 98.5 | S | S | ERR085213 |
| CUGN01 | ncbi | NA | NA | NA | NA | NA |  | NA | NA | 98.5 | R | S | ERR085275 |
| CUEA01 | ncbi | NA | NA | NA | NA | NA |  | NA | NA | 98.4 | S | S | ERR085207 |
| CUGC01 | ncbi | NA | NA | NA | NA | NA |  | NA | 46 | 98.4 | S | R | ERR085266 |
| CUGI01 | ncbi | NA | NA | NA | NA | NA |  | NA | 46 | 98.4 | R | R | ERR085271 |
| CUGP01 | ncbi | NA | NA | NA | NA | NA |  | NA | 49 | 98.6 | R | R | ERR085278 |
| CUFL01 | ncbi | NA | NA | NA | NA | NA |  | NA | 14 | 98.1 | R | R | ERR085250 |
| CUEQ01 | ncbi | NA | NA | NA | NA | NA |  | NA | 11 | 98.4 | R | R | ERR085221 |
| CUDG01 | ncbi | NA | NA | NA | NA | NA |  | NA | 53 | 98.7 | R | R | ERR085188 |
| CUFT01 | ncbi | NA | NA | NA | NA | NA |  | NA | 53 | 98.5 | S | R | ERR085259 |
| CUGA01 | ncbi | NA | NA | NA | NA | NA |  | NA | NA | 98.8 | R | R | ERR085264 |
| CUWC01 | ncbi | NA | NA | NA | NA | NA |  | NA | 8 | 98.5 | R | R | ERR085248 |
| CUFS01 | ncbi | NA | NA | NA | NA | NA |  | NA | 8 | 98.3 | R | R | ERR085253 |
| CUFU01 | ncbi | NA | NA | NA | NA | NA |  | NA | 8 | 98.6 | R | S | ERR085255 |
| CUFQ01 | ncbi | NA | NA | NA | NA | NA |  | NA | 8 | 98.6 | R | S | ERR085254 |
| CUEC01 | ncbi | NA | NA | NA | NA | NA |  | NA | 8 | 98.8 | S | S | ERR085208 |
| CUCL01 | ncbi | NA | NA | NA | NA | NA |  | NA | 8 | 98.3 | S | R | ERR085168 |
| CUER01 | ncbi | NA | NA | NA | NA | NA |  | NA | 2 | 98.6 | NA | NA | ERR085220 |
| NCTC11402 | ncbi | NA | NA | NA | NA | NA |  | NA | 26 |  | S | S | SRR5128002 |

Abbreviations: sex: M (male), F (female); delivery: C (caesarean section), V (vaginal delivery); birth weight in grams, ST: sequence type; OXA, GEN: oxacillin, gentamicin susceptibility test result; R: resistant, S: susceptible. NA: not available.
