# Supplementary figures and images for: Staphylococcus haemolyticus is a reservoir of antibiotic resistance genes in the preterm infant gut
Source: Gut Microbes. 2025 Jun 22;17(1):2519700. doi: 10.1080/19490976.2025.2519700 (PMC12323770; doi:10.1080/19490976.2025.2519700)

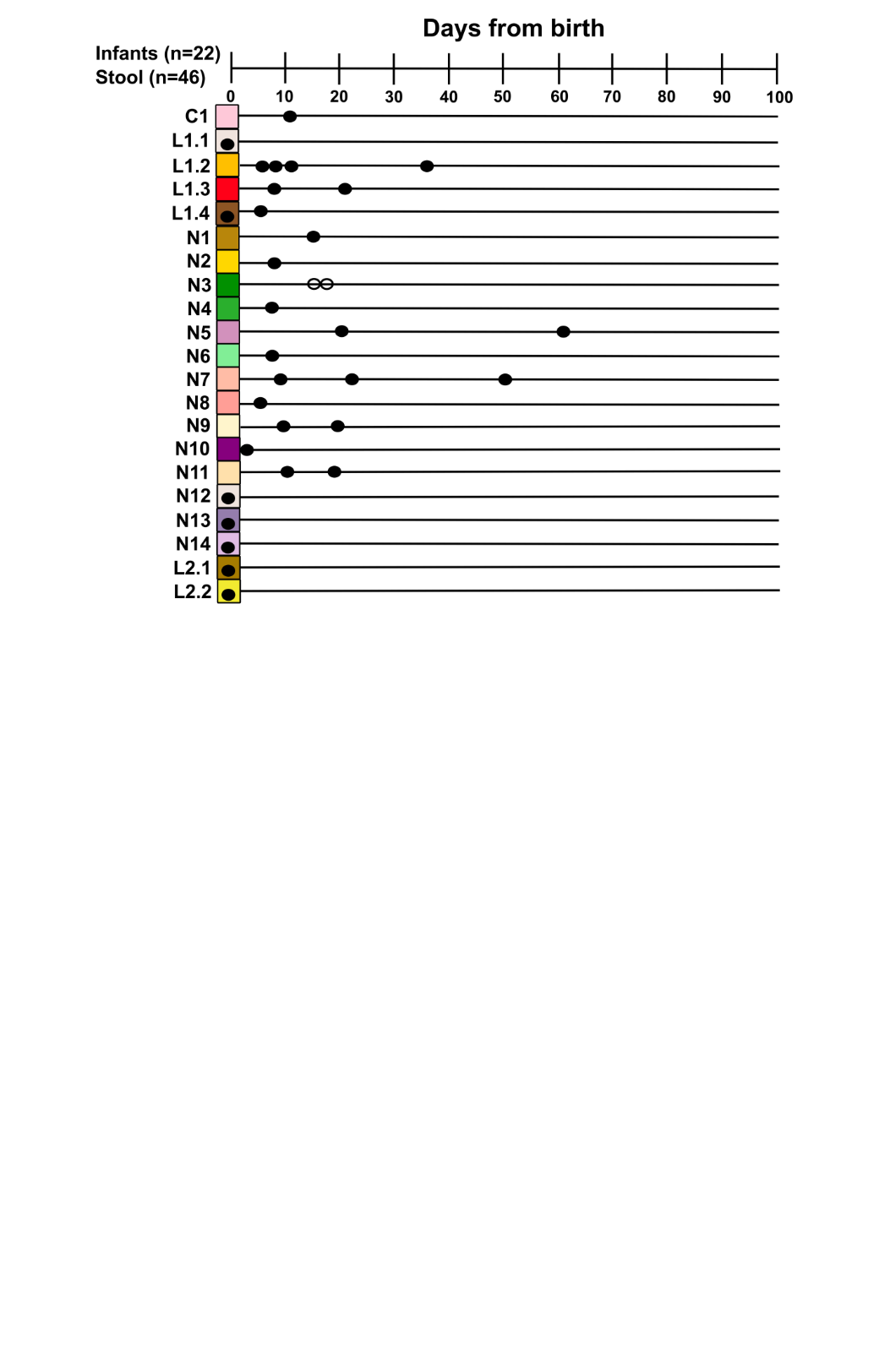


Supp 1


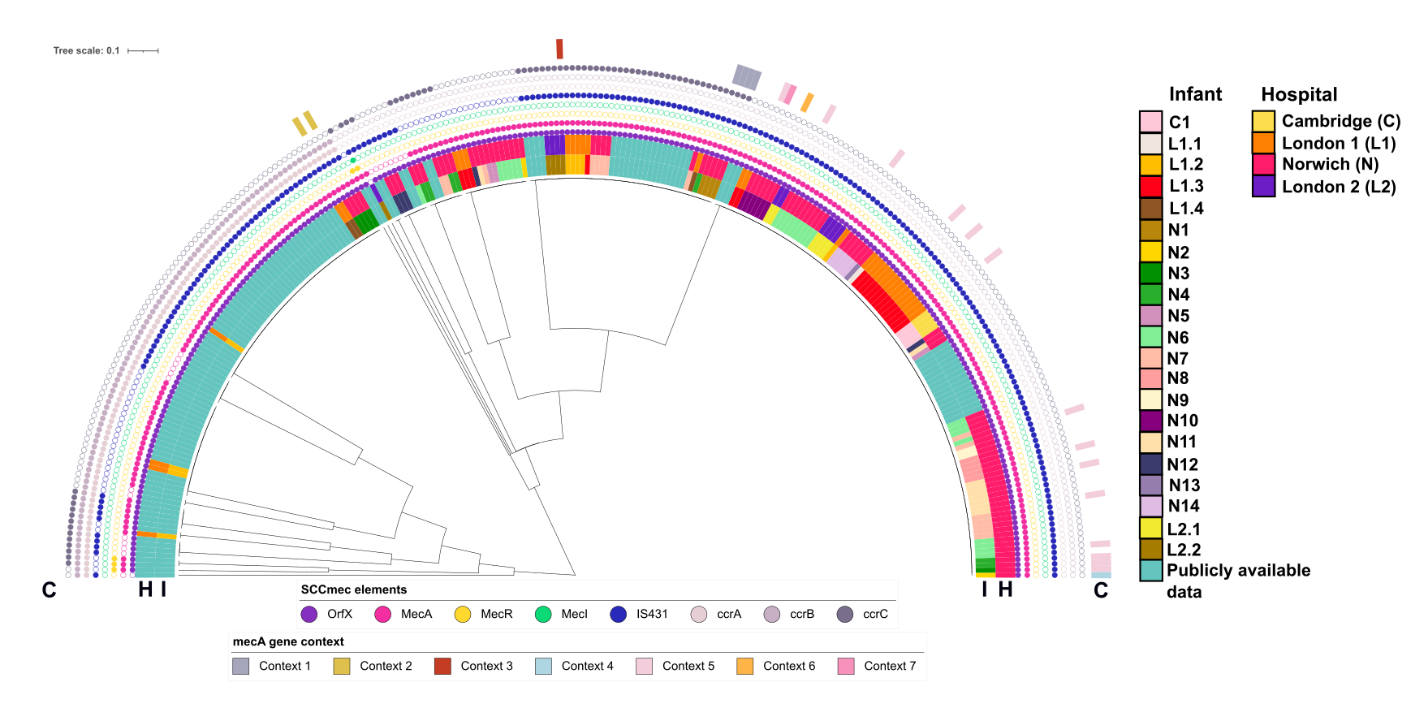


Supp 2


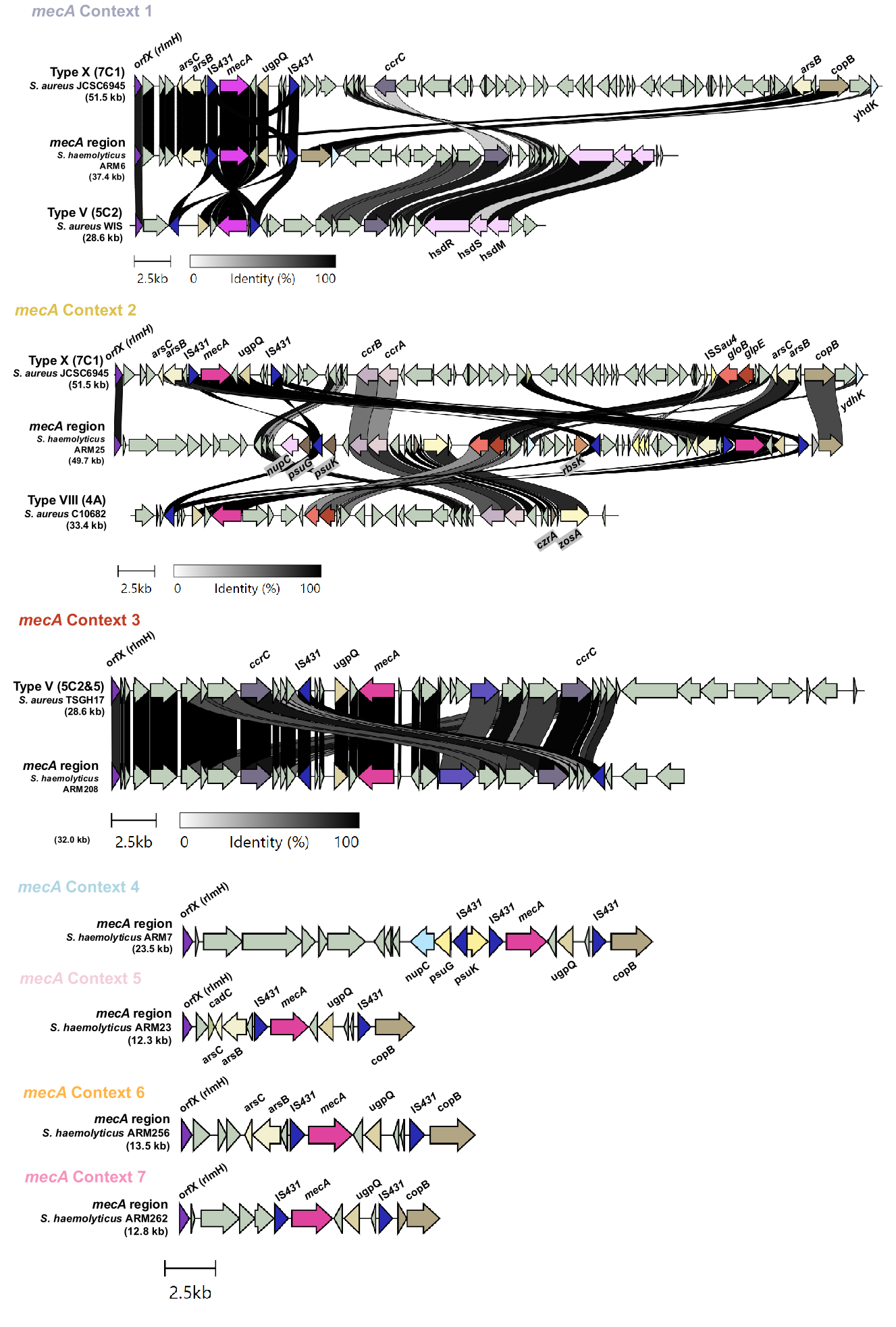


Supp 3


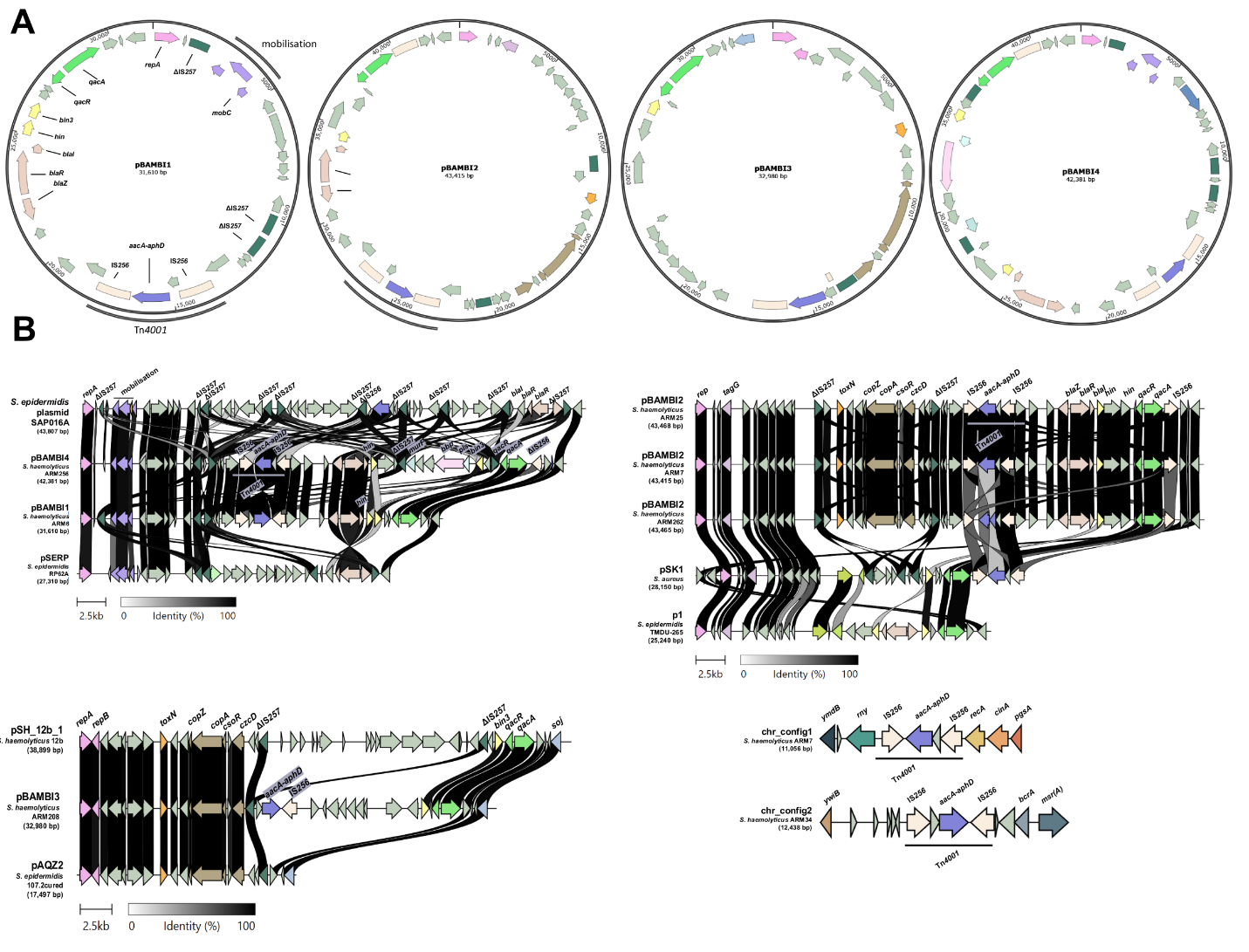


Supp 4

Supplement: Supplemental Material [file KGMI_A_2519700_SM3451.zip › Supp figures.docx]
